# Histone modifications involved in cassette exon inclusions: a quantitative and interpretable analysis

**DOI:** 10.1186/1471-2164-15-1148

**Published:** 2014-12-19

**Authors:** Hui Liu, Ting Jin, Jihong Guan, Shuigeng Zhou

**Affiliations:** Research Lab of Information Management, Changzhou University, 213164 Changzhou, China; Shanghai Key Lab of Intelligent Information Processing, and School of Computer Science, Fudan University, 200433 Shanghai, China; Department of Computer Science and Technology, Tongji University, 201804 Shanghai, China; School of Information Science and Technology, Hainan University, 570228 Hainan, China

**Keywords:** Histone modifications, Alternative splicing, Quantitative analysis

## Abstract

**Background:**

Chromatin structure and epigenetic modifications have been shown to involve in the co-transcriptional splicing of RNA precursors. In particular, some studies have suggested that some types of histone modifications (HMs) may participate in the alternative splicing and function as exon marks. However, most existing studies pay attention to the qualitative relationship between epigenetic modifications and exon inclusion. The quantitative analysis that reveals to what extent each type of epigenetic modification is responsible for exon inclusion is very helpful for us to understand the splicing process.

**Results:**

In this paper, we focus on the quantitative analysis of HMs’ influence on the inclusion of cassette exons (CEs) into mature RNAs. With the high-throughput ChIP-seq and RNA-seq data obtained from ENCODE website, we modeled the association of HMs with CE inclusions by logistic regression whose coefficients are meaningful and interpretable for us to reveal the effect of each type of HM. Three type of HMs, H3K36me3, H3K9me3 and H4K20me1, were found to play major role in CE inclusions. HMs’ effect on CE inclusions is conservative across cell types, and does not depend on the expression levels of the genes hosting CEs. HMs located in the flanking regions of CEs were also taken into account in our analysis, and HMs within bounded flanking regions were shown to affect moderately CE inclusions. Moreover, we also found that HMs on CEs whose length is approximately close to nucleosomal-DNA length affect greatly on CE inclusion.

**Conclusions:**

We suggested that a few types of HMs correlate closely to alternative splicing and perhaps function jointly with splicing machinery to regulate the inclusion level of exons. Our findings are helpful to understand HMs’ effect on *exon definition*, as well as the mechanism of co-transcriptional splicing.

**Electronic supplementary material:**

The online version of this article (doi:10.1186/1471-2164-15-1148) contains supplementary material, which is available to authorized users.

## Background

Nucleosome is the fundamental building unit of eukaryotic chromatin, consisting of ∼147-bp double-helical DNA (nucleosomal DNA) wrapped around a histone octamer in the left superhelix. In all, 75–90% of genomic DNA is packaged into nucleosomes, with adjacent nucleosomes being separated by stretches of DNA, which are referred as linker sequences [1, 2]. Various types of post-translational covalent modifications imposed on the histone tails, including acetylation, methylation, phosphorylation and ubiquitination [3, 4], have been shown to play important roles in various biological processes, such as transcription regulation, co-transcriptional splicing, DNA replication and repair, by functioning alone or jointly to change the charge of the nucleosome particle, and/or by recruiting other protein effectors [5–7].

With the advent of genome-wide ChIP-chip and RNA-seq techniques, mapping of global patterns of nucleosome positioning and epigenetic modifications has become commonplace and has been performed on many organisms [8–11]. One insight revealed by such large-scale biological datasets is that exons are marked with high nucleosome-occupancy levels in contrast to introns, and the occupancy pattern of nucleosomes is independent of the gene expression level [12]. The nucleosome distribution difference can not be completely explained by GC content difference between exon and intron [13]. Therefore, nucleosome positioning has been supposed to be finely regulated by transcription machinery and chromatin remodeling complexes to assist the recognition of splicing sites. The concept of exon/intron definition was accordingly proposed to describe the function of nucleosome positioning in RNA precursor splicing [13–15]. Also, it has been shown that alternative splicing is a major mechanism for promoting transcriptome and proteome diversity, particularly in mammals [14]. As indicated by Wang *et al.*, 92–94% of human genes undergo alternative splicing (AS) [16]. Eight type of AS, including skipping exon (ES), mutually exclusive exon (ME), alternative 5’ splice site selection (A5SS), alternative 3’ splice site selection (A3SS) and intron retention (IR), Alternative first exon (AFE), Alternative last exon (ALE) and Tandem 3’ UTRs, have been identified from 15 diverse human tissue and cell line transcriptomes by deep sequencing [16]. Many studies have been conducted to explore the cross-talk between HMs and splicing machinery [5, 17, 18], and found that some type of HMs have high enrichments on exons rather than on introns [12, 19]. For example, H3K36me3 has been suggested to be a conserved epigenetic mark for exons, as it shows significant high level on expressed exons than on introns across many species and cell types [20, 21]. H3K9me was found to function jointly with the chromodomain protein HP1 *γ* to favor inclusion of alternative exons [5]. Meanwhile, some other type of HMs, such as H3K79me1, H4K20me1, have also been shown to be highly enriched in internal exons than in flanking introns, implying that HM patterns are closely associated with the co-transcriptional splicing of RNA precursor [19, 22]. Luco *et al.* have demonstrated that different HM patterns led to different splicing outcomes in a set of human genes [23]. Moreover, HMs have been supposed to participate in multiple type of alternative splicing [24]. Enroth *et al.* trained a rule-based model to predict exon inclusions with considerable accuracy by using HMs proximal to cassette exons (CEs). The generated rules in the form of “*IF …THEN…*” imply that some specific combinational patterns of HMs facilitate exon inclusion while some other patterns inhibit exon inclusion [25]. Zhu *et al.* modeled the quantitative relationship between HMs and CE inclusion by linear regression and showed that HMs were predictive of exon inclusion levels [26].

According to the inventory of known alternative isoforms [16], skipping exons are the most prevalent in mammals among eight types of alternative splicing events, and are mainly responsible for the proteome diversity. In this paper, we therefore focus on the quantitative analysis of which HMs affect the CE inclusion and to what extent HMs affect the CE inclusion, by integrating the ChIP-seq and RNA-seq high-throughput data of three human cell types of ENCODE Tier 1, Gm12878, H1-hESC and K562. More precisely, we regarded respectively the CE inclusion and surrounding HMs as response variable and explanatory variables, and then used logistic regression to model their quantitative relationship. The remarkably high prediction accuracy of CE inclusion based on HMs proximal to the splicing sites confirms previous qualitative conclusions that HMs involve in co-transcriptional splicing. We also showed that comparable accuracy can be obtained by using only a few types of HMs to build the logistic regression models, which implies that several types of HMs dominate the effect on CE inclusion, and their effects were further demonstrated to be independent of the expression levels of the genes hosting the CEs. HMs located in the flanking regions of CEs were taken into account in our analysis, and we found that HMs within bounded flanking regions (about three tandem nucleosomes away from the CE splicing sites) affect moderately CE inclusions. Interestingly, HMs located on CEs whose length are close to nucleosomal DNA show most predictive of CE inclusion. Considering the fact that the average length of human cassette exons is about 150 bp, we thus suppose that exons evolutionarily fit to the nucleosomal-DNA length so that exons exactly wrap the histones, which in turn facilitate epigenetic modifications to participate in co-transcriptional splicing.

## Results and discussion

### Nucleosome positioning is independent of cassette exon inclusion

**Figure 1:**
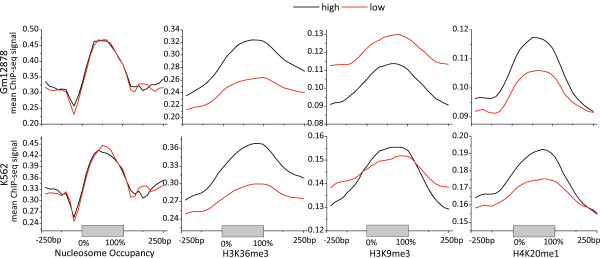
**Distribution of mean ChIP-seq signals of nucleosome and three types of HM, including H3K36me3, H3K9me3 and H4K20me1, on CEs and ±250 bp flanking regions.** The ChIP-seq signals were averaged over each portion of CE samples, which were partitioned into high and low portions according to inclusion levels. The upper and lower half parts correspond to the signal distributions for Gm12878 and K562, respectively.

Many studies have investigated the occupancy pattern of nucleosomes along gene body, and have shown that exons are more considerably marked with high level nucleosome than introns [12, 13, 19]. We here pay attention to the effect of nucleosome positioning on the inclusion level of CEs by inspecting the nucleosome occupancy signal distribution on CEs and flanking regions. We extracted CE samples from the alternative splicing catalog provided by Wang *et al.* [16], and calculated the CE inclusion levels by using MISO [27] from high-throughput RNA-seq data downloaded from ENCODE [28]. MISO outputs the “percentage spliced in” (PSI or *ψ*) that denotes the fraction of mRNA isoforms including CEs of interest. All CEs were partitioned into two portions according to inclusion level by the cutoff *ψ* = 0.5, i.e., CE samples whose inclusion levels were greater than 0.5 were partitioned into the high portion, and the remaining ones were partitioned into the low portion. For simplicity, we refer them as high-class and low-class CEs, respectively. For Gm12878, there were 9266 and 5498 CE samples in the high-class and low-class, respectively. For K562, there were 10993 and 6451 CE samples in the high-class and low-class, respectively. The nucleosome positioning ChIP-seq data of H1-hESC cell line is not available on ENCODE, so its distribution of nucleosome positioning is absent. Nucleosome occupancy signals were mapped to CEs and ±250 bp flanking regions, and then the mean occupancy levels were calculated over all CE samples of each class. The left column of Figure 1 shows the distribution of nucleosome occupancy of the two cell types Gm12878 and K562. Consistent with previous studies, nucleosome level is remarkably high on CEs compared to flanking introns, and a nucleosome free region is observed near the 3’ splice sites. However, the most noteworthy is that there is no significant difference of nucleosome level among the two classes of CEs with different inclusion levels (paired sample Wilcoxon signed rank test, *p*-values were 0.147 and 0.416 for Gm12878 and K562, respectively). We thus suggests that nucleosome positioning is independent of CE inclusion level, we will go further to validate the suggestion by logistic regression of CE inclusion with respect to the nucleosome positioning signals in the following context. The difference of nucleosomes levels between exons and introns is more like a stationary feature that is either endogenously encoded by sequence affinity to histone octamers, or is maintained by chromatin remodeling complexes to facilitate the recognition of splice sites by splicing factors.

### Histone modifications correlate closely to cassette exon inclusion

We inspected whether HMs associate with the inclusion level of CEs. For the three types of cell lines, we derived the ChIP-seq signal distributions of all types of HMs on CEs and ±250 bp flanking regions in the similar way mentioned above. The 2–4th columns of Figure 1 showed the mean ChIP-seq signal distributions of the three types of HMs, H3K36me3, H3K9me3 and H4K20me1, for Gm12878 and K562 cell lines, Additional file 1: Figure S1 showed the mean ChIP-seq signal distributions of other seven type of HMs for Gm12878 and K562, and Additional file 1: Figure S2 showed the signal distributions of all type of HMs for H1-hESC cell line available on ENCODE. From these signal distributions, it can be found that the HM signals corresponding to the two different inclusion levels of CEs diverges greatly in comparison to nucleosome positioning signals. H3K36me3 showed positive correlation to CE inclusion levels, i.e., the higher the CE inclusion levels, the stronger the signals on both CEs and flanking regions. H4K20me1 display similar signal distributions to H3K36me3, although its divergence between two classes was not as large as that of H3K36me3. H3K9me3 and H3K27me3 display slightly different distributions, the mean ChIP signals on CEs of high-class CEs is stronger than that of the low-class, while the contrary is the case on the flanking regions. Other type of HMs, including H3K9ac, H3K27ac, H3K4me3, H3K4me2 and H3K79me2, showed significantly different signal distributions that were negative correlated to CE inclusion levels, i.e., the low-class had stronger signals compared to the high-class. Interesting, these HMs can be categorized into two types in the view of their signal curve shape: bell and slash. As HMs on flanking regions have also been reported to affect CE inclusion, we regarded the HM signals of upstream and downstream regions ±250 bp flanking CEs as independent variables, and thus each type of HM corresponds to three components, denoted by suffix _up, mid and _down, respectively. We calculated the Pearson correlation coefficients between each pair of variables and performed hierarchal clustering, as shown in Additional file 1: Figure S3. Also, the correlation coefficients between CE inclusion level (represented as miso_level) and HMs were also shown in Additional file 1: Figure S3. It is found that the three components of each type of HMs positively correlated to each other. Also, H3K27ac, H3K9ac, H3K4me2, H3K4me3 and H3K79me2 were positively correlated to each other and thus formed a dense cluster, while H3K36me3, H3K9me3, H3K27me3 and H4K20me1 diverged from other HMs and formed another relatively loose cluster. In particular, we found that H3K36me3_mid and H3K36me3_down had considerably positive correlation coefficients to CE inclusion level. Take Gm12878 as an example, the correlation coefficients were 0.21 (p-value = 3.7e-148) and 0.143 (p-value = 3.1e-68). In addition, H4K20me1_mid also had positive correlation coefficients to CE inclusion level for all three cell lines, while H4K20me1_down and H3K9me3_down showed negative correlation. In summary, HMs show considerably divergent signal distributions corresponding to different CE inclusion levels, especially compared to the nucleosome positioning signal distribution, we therefore suggested that some types of HMs, such as H3K36me3 and H4K20me1, on CEs and flanking regions were closely related to CE inclusion.

### Modeling CE inclusion by Logistic regression

We go one step further to computationally model the correlation between HMs and CE inclusion. The distributions of CE frequencies with respect to the binned inclusion levels were derived based on the *ψ* values for the three cell lines, as shown in Additional file 1: Figure S4. We found that the the inclusion levels of most CEs are close to either 0 or 1, and the cumulative frequency distributions approach to the CDF of a specific Bernoulli distribution, indicating that most CEs are universally either included into or excluded from mature RNAs in the measured cell population. It is rational for us to suppose that the inclusion levels of CE samples were generated from a binomial distribution, given that sequencing errors and the ambiguity of mapping short reads to genome are responsible for some noise samples.

Therefore, we regarded the inclusion of CEs as a two-class classification problem, for which a variety of classifiers can be applicable. For the sake of interpretability, we employed the logistic regression model to estimate the effect of each type of HMs on the inclusion of CEs, as its regression coefficients and odd-ratios are meaningful for evaluating the importance of each explanatory variable. Specifically, we regarded the signals of each type of HMs on CEs as an explanatory variables, and the CE inclusion as a binary response variable. HM signals on upstream and downstream regions ±250 bp flanking CEs are also respectively regarded as independent explanatory variables. For each cell line, we took ten types of HMs that expanded to 30 explanatory variables to build the logistic regression models. The inclusion levels were discretized by applying a predefined cutoff *δ* (0.5 ≤ *δ* < 1), so that the inclusion levels greater than *δ* were set to 1 and the inclusion levels less than 1-*δ* were set to 0.

**Figure 2:**
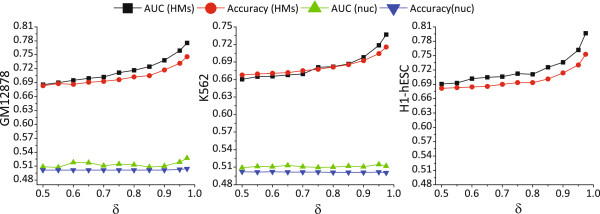
**AUC and accuracy curves of the logistic regression models built based on respective the nucleosome positioning and HM signals on CE and ±250 bp flanking regions for three cell lines.** For H1-hESC, the curves corresponding to nucleosome positioning are absent because the nucleosome ChIP-seq signal is not available on ENCODE.

Figure 2 showed the AUC curves of the logistic regression models, together with the accuracies upon independent test sets, corresponding to different *δ* values for CE inclusion level discretization of the three cell lines. For Gm12878 and H1-hESC, AUC values of the models built based on HMs were always greater than about 0.68, increased gradually and approached to 0.79 when *δ* increased from 0.5 to 0.975. The accuracy curves followed the same trend to the AUC curves, increased from about 0.68 to 0.75. K562 had the similar AUC and accuracy curves, by ∼3*%* difference compared to Gm12878. The results demonstrate that HMs are highly predictive of CE inclusions. We strongly suggest that HMs involve directly or indirectly in the co-transcriptional splicing process, although the functional mechanism is unclear yet. Besides, we found that AUC and accuracy curves kept steady when *δ* was no more than than 0.85. For all the three cell lines, about 80% CE samples (>10,000 samples) were retained when *δ* is set to 0.85. So, we thereafter set *δ* to 0.85 for the following experiments.

It is helpful to reinspect the relationship between nucleosome positioning and CE inclusions by applying the logistic regression model. Therefore, we built logistic models based on only nucleosome signals using the same way mentioned above. The AUC and accuracies of the models built on nucleosome positioning signals were also shown in Figure 1 for Gm12878 and K562 (H1-hESC’s nucleosome positioning is not available). Both ROC and accuracy curves were very close to 0.5 and kept almost unchanged when *δ* increased from 0.5 to 0.975, implying that the prediction accuracy was roughly equal to random guess. The results confirm our previous conclusion that nucleosome positioning is independent to CE inclusion.

### HMs are predictive of differentially expressed CEs

We are interested in whether HM signals are predictive of the differentially expressed CEs across the three cell lines, which motivate us to check whether the logistic regression model built on one cell line is predictive of the inclusion of CEs that are differentially expressed in another cell line. For this purpose, we employed MISO to detect the differentially expressed CEs by setting the parameter *Bayes_factor* to 2, which would filter out the CE samples that were two times more likely to be differentially expressed CEs than not (for more detail see Methods). Take Gm12878 vs. K562 as an example, 963 differentially expressed CE samples were filtered out. We trained the logistic regression model based on the these differentially expressed CE samples of Gm12878 and then used the trained model to predict the CE inclusion of K562, and vice versa. We thus have six cross-comparison experiments for the three cell lines. The numbers of differentially expressed CE samples and AUCs were shown in Table 1. We can found that the models built on one cell line were significantly predictive of the CE inclusions that were differentially expressed in another cell lines, although the prediction accuracy decreased slightly compared to that on the same cell line. The results imply that the predictability of CE inclusions by HMs is not cell type-specific, but conservative across cell types.

**Table 1:** **Number of differentially expressed CE samples (#CEs) and AUC values of the logistic regression models for predicting differentially expressed CE inclusion across cell lines**

			Cell line for testing			
Cell line for training	Gm12878		K562		H1-hESC	
	#CEs	AUC	#CEs	AUC	#CEs	AUC
Gm12878			963	0.677	613	0.668
K562	963	0.659			608	0.663
H1-hESC	613	0.647	608	0.678		

### Several types of HMs play dominant role on regulate CE inclusion

To estimate the effect of each type of HM on CE inclusion, we inspected the regression coefficients and odds-ratios obtained by logistic regression. Additional file 1: Figure S5 illustrated the regression coefficients and odds ratios on natural-log scale for Gm12878. Overall, H3K36me3, H3K9me3, H4K20me1 and H3K27me3 possessed significant regression coefficients and log-scaled odds-ratios compared to the others type of HMs. Especially, H3K36me3_mid and H3K36me3_down have large positive regression coefficients and odds-ratios, which implies the dominant role in CE inclusion played by H3K36me3. This is consistent with a variety of previous studies that H3K36me3 marks expressed exons [20, 21]. For the K562 and H1-hESC cell lines, we got similar results that H3K36me3, H3K9me3 and H4K20me1 still hold the top three large regression coefficients, as shown in Additional file 1: Figure S6-S7. In addition, it is worth noting that H3K9me3_mid in H1-hESC achieved even larger regression coefficient and log-scaled odds-ratio than H3K36me3_mid and H3K36me3_down. This is consistent with another straightforward evidence that H3K9me3 participates in the inclusion of alternative exons by functioning jointly with chromodomain protein HP1 *γ* [5]. H3K9me3_down, H4K20me1_down and H3K27me3_down had large negative regression coefficients and log-scaled odds-ratios, which indicated that the HMs succeeding the CEs function to inhibit CE inclusion. In fact,the inhibitive function on CE inclusion of these downstream HMs has also been suggested by Enroth *et al.* [25]. This is also consistent with Podlaha et al.’s conclusion that HMs correlated to CE inclusion via specific spatial patterns along the upstream and downstream regions around CEs [29]. Although no biologically experimental evidence supporting the function difference played by HMs located relatively different positions have been proposed, we suppose that these *in silico* results provide a strong hint for biochemical validation.

Furthermore, we took a look inside the model selection process of logistic regression in which each explanatory variable was progressively added into the model learned so far to search for the best model. For each step, a likelihood score (chi-square test) was calculated to evaluate the model when a new variable is added. Intuitively, the priority of a variable being selected into the current model indicates that this variable carries more information about the response variable than other unselected variables. The likelihood score curves for the three cell lines were shown in Additional file 1: Figure S8, from which we found that the chi-square score increases rapidly at the beginning and then tends to steady after about 10 variables were included in the model. Therefore, we considered the top 10 models for each randomly generated training set, and got the frequency of each variable after 50 times of sampling and training processes, as shown in the left part of Additional file 1: Figure S9-S11. The three components of H3K36me3, H3K9me3 and H4K20me1 were found to possess dominant frequencies in contrast to those of the other HMs. If we sum up the frequencies of the three components corresponding to each type of HM, we thus got the frequency distributions over all types of HMs, as shown in the right part of Additional file 1: Figure S9-S11 for the three cell lines. In fact, Karlic *et al.* also adopted such method to estimate the importance of each HM on gene expression levels [30]. It was clear that H3K36me3 achieved the highest frequency, followed by H3K9me3, H4K20me1. Based on the observations above, we speculate that a few types of HMs, such as H3K36me3, H3K9me3 and H4K20me1, play dominant roles in CE inclusion. To confirm our hypothesis, we built three logistic regression models by successively using the component combinations belong to these HMs: H3K36me3, H3K36me3+H3K9me3, H3K36me3+H3K9me3+H4K20me1, which were referred as three simplified models in the following context. In contrast, the model included all HMs was referred as full model. As illustrated in Table 2, the AUCs and accuracies of the three simplified models, especially the model H3K36me3+H3K9me3+H4K20me1, were comparable to those of the full model. On the contrary, if we remove the three types of HMs and build the logistic regression model based on the remaining HMs, the performance deteriorated dramatically and AUCs were no more than 0.6 on all the three cell lines, as shown in Additional file 1: Figure S12. These results confirm our hypothesis that only a few types of HMs play dominant role in the regulation of CE co-transcriptional splicing.

**Table 2:** **AUC values and accuracies of the logistic regression models built on the three simplified and full model**

Variables in model	Measure	Gm12878	K562	H1-hESC
H3K36me3	AUC	0.68769	0.66035	0.70074
	Accuracy	0.66372	0.65873	0.67458
H3K36me3+H3K9me3	AUC	0.70985	0.67759	0.71738
	Accuracy	0.67382	0.66972	0.68834
H3K36me3+H3K9me3	AUC	0.71418	0.69225	0.7207
+H4K20me1	Accuracy	0.69024	0.67639	0.70843
Full model	AUC	0.72812	0.69831	0.73418
	Accuracy	0.71885	0.68092	0.71069

### HM’s effect on CE inclusion does not depend on gene expression level

The elongation speed of RNA Pol II has been suggested to affect CE inclusion and shape the pattern of HMs [31, 32]. Slow speed of RNA Pol II elongation nearby splicing sites is helpful for exon recognition by splicing factors and thus favors exon inclusion [14]. This inspires our interest in whether the effect of HMs on CE inclusion is correlated to the expression level of hosting genes. For this purpose, we calculated the number of Fragments Per Kilobase of exon per Million fragments mapped (FPKM) by cufflinks [33]. FPKM was first proposed by Mortazavi *et al.* [34] and has been widely used as the gene expression levels by many studies [25, 27], as they have higher sensitivity and accuracy than microarray signals. In our analysis, FPKM values were normalized into range [0,1], and then mapped to CEs by virtue of the annotation file that links CEs to their hosting genes [27].

**Figure 3:**
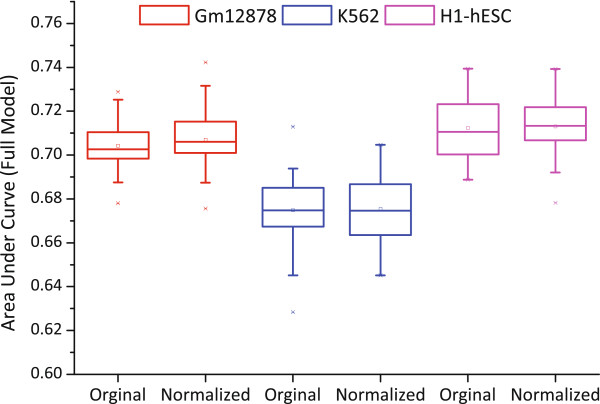
**AUC boxplots of logistic regression models built on original HM signals and normalized HM signals by FPKMs of hosting genes, respectively.** There is no significant difference of the AUCs before and after signal normalization for all three cell lines.

We first inspected the correlation between CE inclusions and the expression levels of hosting genes. The Pearson correlation coefficient is only 0.03577 (p-value < 0.0001). We went further to check whether the predictability of CE inclusions by HMs was an implicit result of transcriptional regulation of gene expression. For this purpose, we eliminate the effect of hosting gene expression level on HM signals, by performing linear regression for HM signals with respect to FPKM values of the hosting genes, and got the residuals of each type of HM. The residuals was subsequently used to build the logistic regression models. As shown in Figure 3, the AUC values derived from residual signals are very close to those derived from the original signals in all three cell lines. Take Gm12787 as an example, the null hypothesis that the AUC mean values between original and normalized signals is equal is not be rejected by *t*-test (*p*-value = 0.243). Moreover, We ranked all CE samples according to FPKM values and partitioned them into five parts, referred as lower, low, median, high and higher training sets, respectively. For each part, we independently built the logistic regression models and show the AUC values in Additional file 1: Figure S12. Neither meaningful pattern from the AUC curves nor significant performance divergence can be found among the five subsets of CE samples. In particular, the largest AUC difference among the five training sets of the three cell lines is no more than 0.03. In all, these results imply that HM’s effect on CE inclusions are negligibly affected by the expression levels of hosting genes.

### HMs in flanking regions have only limited effect on CE inclusion

**Figure 4:**
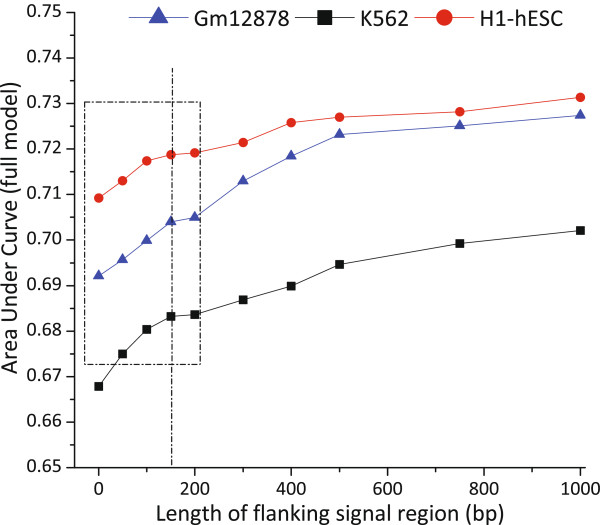
**AUC curves of the logistic regression models built based on HM signals located on increasing-size flanking regions for three cell lines Gm12878, K562 and H1-hESC, respectively.**

Some studies have reported that HMs in the regions flanking splicing sites affect CE inclusion [25, 29]. We are curious about how HMs influence CE inclusion when they locate farther and farther away from the CE boundaries. For this purpose, we built multiple logistic regression models with respect to HMs located in varying-size flanking regions. In particular, HM signals in ten different sizes of flanking region, including 0 bp, 50 bp, 100 bp, 150 bp, 200 bp, 300 bp, 400 bp, 500 bp, 750 bp and 1000 bp, were respectively used to build the models. Figure 4 shows the AUC values of the logistic regression models learned from the signals of different-size flanking regions for the three cell lines. Overall, the AUC curves increased considerably at first with the increasing size of flanking region, then go up gradually and finally become nearly stable when the size exceeds 500 bp (about the length of three tandem nucleosomes). Particularly, a closer look showed that the AUC curves form broad peaks at ∼160 bp that is approximately equal to the length of the DNA wrapped around a nucleosome (∼147 bp) plus the linker DNA, as illustrated in the dashed line box in Figure 4. We thus suggest that the HMs closer to CE boundaries, especially the nucleosome proximal to CE boundaries, have stronger effect on CEs inclusion than those nucleosomes located farther from the CE boundaries. On the other hand, it should be noted that the accuracy is limitedly improved (about 4%) even when the size of flanking regions extended to 1000 bp. If only the HM signals on flanking regions were taken into account, i.e., the HMs located on CE were excluded from building the models, the performance declined dramatically but still outperform random guess, as shown in Additional file 1: Figure S13. Taken together, these results imply that HMs located in bounded flanking regions have statistically significant but limited effect on CE inclusion.

### HMs on approximate nucleosomal DNA-length CEs greatly affect CE inclusion

**Figure 5:**
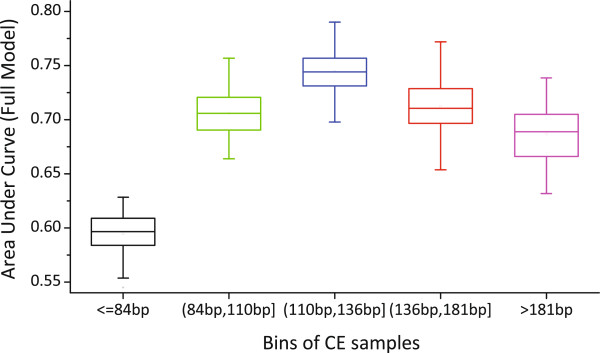
**AUC boxplots of the logistic regression models learned from five subsets of CE samples independently for Gm12878.** All CEs were ranked in ascending order according to exon lengths and then split into five bins by equal-depth partitioning rule.

**Figure 6:**
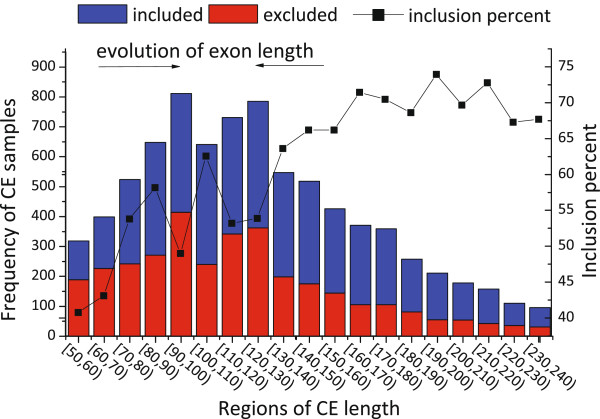
**Histogram of the CE frequencies regarding exon length for Gm12878, together with the percent of CEs included in mature RNAs.**

We proceed to investigate the relationship between the CE length and inclusion level. All CE samples were sorted according to their lengths, and then were binned into five bins by the equal-depth partitioning rule, i.e., each bin contained the same number of CE samples. For Gm12878, we got four split points of the CE length, which were 84 bp, 110 bp, 136 bp and 181 bp, and each bin contained about 2952 CE samples. For each bin, we built the logistic regression model as the same method mentioned above. Figure 5 illustrates the AUC boxplots of the logistic regression models independently built on the five bins of CEs for Gm12878. We found that the first bin whose CE length was no more than 84 bp has remarkably lower AUC value than the other bins. The third and fourth bins, whose CEs have lengths closest to the length of nucleosomal DNA (∼147 bp), achieve higher AUC values than other bins. This result implies that CE length affect greatly HMs’ function on CE inclusion. Furthermore, we got the CE frequency distribution over CE length for Gm12878 and found that the lengths of most CEs falls into the range of 100 bp-160 bp (75%), as demonstrated in Figure 6. It can be also found from Figure 6 that the CE inclusion level is positively correlated to CE length. This is consistent with previous findings that long exons have high inclusion levels compared to short exons [15, 35]. Another consistent fact is that the average length of human cassette exons is 146 bp (very close to the nucleosomal DNA length). Similar results were obtained for K562 and H1-hESC, as shown in Additional file 1: Figure S14-S17. In summary, we derive an intuitive explanation that exons evolutionarily fit to the nucleosomal-DNA length so that exons exactly wrap the histones, which perhaps facilitates a series of subsequent splicing processes, including the RNA polymerase pausing at splicing sites, exon recognition and spliceosome assembly. As a result, HMs exerted on histone tails are naturally exploited by these protein factors, or vice versa, i.e., splicing machinery dynamically modulates HMs to aid subsequent splicing events in a “reaching back” way [31, 32]. While those exons in the first bin are too short to wrap around the histones to form stable nucleosomes, leading to the consequence that HMs on these short exons are less functional in splicing regulation.

## Discussion

RNA precursor splicing and transcription elongation are traditionally deemed to function separately, but increasing evidence suggests that splicing is functionally coupled to transcription elongation [36]. It has been proposed that specific HMs may help to recruit related TFs or splicing factors, or to change the RNA Pol II elongation speed, which can affect the assembly of spliceosomes or the recognition of splicing sites [12, 19]. However, few studies have paid attention to the quantitative relationship between HMs and exon inclusions. In this paper, we used high-throughput ChIP-seq and RNA-seq data covering three human cell lines to carry out a comprehensive quantitative analysis, and found that HMs along exons and flanking regions are strongly predictive for exon inclusions.

Consistent to previous studies [19, 20, 23], H3K36me3 was found to be most predictive for CE inclusions in our analysis. In particular, [23] utilized biological experiments (RNA interference, RNA immunoprecipitation and quantitative RT-PCR) to validate the causal role of HMs in alternative splice site selections, and indicated that H3K36me3 could interact with PTB protein to regulate alternative splicing. The observations validate our findings. H3K9me3 was also shown to play considerable effect on exon inclusions, which has been demonstrated to favor exon inclusion by involving the recruitment of HP1 *γ* protein [5]. It is worth to note the bidirectional functions of H3K9me3, i.e., its high level on promoter inhibits gene expression while its enrichment on gene body favors exon inclusion. In addition, H4K20me1 has been suggested to involve exon inclusion by several previous works [14, 24, 25] and our analysis. Although the mechanism of H4K20me1 affecting exon inclusion has not been experimentally confirmed, our analysis may indicate its genuine role and help planning biochemical experiments.

Some researchers have revealed the quantitative correlation between HMs on promoter and gene expression level. For example, causal relationship between HMs and gene expression has been inferred by building Bayesian network [37]. Karlic *et al.* [30] showed the high prediction accuracy by modeling the relationship between HMs and gene expression level using simple linear regression. Furthermore, HMs on transcribed regions have been shown to be more predictive for gene expression level than those on promoters [26]. It seems that the predictability of HMs for exon inclusion should be affected by gene expression level. But our analysis suggests that the predictability is independent of the expression level of hosting genes. Moveover, HMs on exons are dominantly predictive compared to those on flanking regions, although the later can slightly improve the prediction accuracy.

However, some studies have indicated that the exon-intron marker of HMs is cell type-specific, and even some HMs patterns show bias towards introns [38]. Stable HM profiles have been observed even if different splicing results are induced by RNA interference [26]. Furthermore, the causality between HM patterns and exon inclusions has not been specified. In fact, both directions of causality have been reported. HMs have been shown to interact with splicing factors to influence alternative splicing [23], while a recent study indicated that specific HMs could be determined by pre-mRNA splicing [20]. These findings confound the emerging concept of *exon definition* by HMs. Actually, the co-transcriptional splicing of RNA precursor is a complex and multi-step biological process that involves recognition of splice sites, spliceosome assembly and intron excision. HMs were suggested to participate in one or more of the processing steps, resulting in the establishment of specific HM patterns. For example, the dynamic change of histone acetylation and deacetylation has been shown to drive the spliceosome assembly and rearrangement [32]. On the contrary, Zhou *et al.* have demonstrated that splicing regulators interact with histone modifying enzymes to modulate the chromatin structure for the sake of proper transcriptional elongation rate [31]. The causality of HMs marking exons, i.e., HM patterns act as the role for recognition of splice sites or HM patterns are just the result of spliceosome activities, is not clear. More studies should be devoted to exploring the interacting mechanism of HM with splicing machinery.

Finally, it is worthy to point out that our analysis has some common findings with Zhu *et al.* in quantitatively modeling CE inclusion by HMs. Both our analysis and Zhu *et al.* found HM signals around CEs were predictive of CE inclusion level, and the quantitative correlation is conservative across cell types. But our analysis differed from Zhu *et al.*’ findings in at least three aspects [26]. First, we show that HMs’ effect on CE inclusion do not depend on gene expression level. Second, we analyzed the the effect of HMs located in flanking regions, and found that HMs within bounded flanking regions moderately affect CE inclusion, while Zhu *et al.* overlook this important phenomenon. Third, we explored the relationship between exon length and CE inclusion, and found that HMs on approximate nucleosome-length exons affect mostly on CE inclusion. Besides, the sequencing depth of the ChIP-seq and RNA-seq data of the ENCODE datasets used in our analysis is far larger than that used by Zhu *et al.*, which enable us to mine more deeply to the epigenetic regulation.

## Conclusions

In this paper, we carried out a comprehensive quantitative analysis of HMs’ effect on CE inclusion by integrating the ChIP-seq and RNA-seq high-throughput data of three human cell lines, Gm12878, H1-hESC and K562. We employed the logistic regression model to capture the quantitative relationship between HMs and CE inclusion, and obtained significant prediction accuracy based on the HMs located on CEs and flanking regions. We also showed that considerably high accuracy can be obtained by using only a few types of HMs, including H3K36me3, H3k9me3 and H4K20me1, to learn logistic regression models, which implies that several types of HMs dominate the effect on CE inclusion. We went further to demonstrate that HMs’ effect on CE inclusion is conservative across cell tpye and does not depend on the expression levels of the genes hosting the CEs. Furthermore, HMs located in the regions flanking CEs were also taken into account in our analysis, and were shown to had statistically significant but limited effect on CE inclusions. Interestingly, HMs on CEs whose lengths were close to nucleosomal DNA showed most predictive of CE inclusion. Based on the findings we concluded that HMs on CEs and flanking regions function jointly with splicing machinery to regulate the RNA precursor splicing.

## Methods

We chose the three human cell lines of Tier 1 cell types of ENCODE [28], Gm12878, K562 and H1-hESC, because both the CE annotations and high-throughput sequencing data (ChIP-seq and RNA-seq) are available, which facilitates us to carry out quantitative studies.

### ChIP-seq and RNA-seq data

High-throughput ChIP-seq datasets of HMs tracks published by Broad Institute of MIT and Harvard were employed in our analysis [39]. For each cell line, only ten type of HMs, including H3K36me3, H3K9me3, H4K20me1, H3K27ac, H3K9ac, H3K4me2, H3K4me3, H3K79me2, H3K27me3 and H3K4me1, were taken into account. Other protein factors, such as H2A.Z, RNA Pol II, etc. were excluded. Short reads were aligned to the human reference genome (GRCh37/hg19) using MAQ [40] with default parameters, and fragment densities were computed by counting the number of reads overlapping each 25 bp window along the genome.

ChIP-seq datasets of nucleosome positioning tracks [41] for Gm12878 and K562 provided by Stanford University were obtained. Short reads were mapped to the human reference genome (GRCh37/hg19) in color-space with the probabilistic mapper, DNAnexus. Nucleosome density signals were generated by first shifting reads by 74 bp in the 5’ to 3’ direction and then counting the total number of reads starting at each genomic coordinate on both strands. These counts were then smoothed using a 60 bp window.

Pair-end RNA-seq tracks provided by California Institute of Technology (Caltech) were used for our analysis [42]. Raw reads (2 × 75 bp) were mapped to the reference human genome (version hg19) using TopHat [43] with default settings, with the exception of specifying an empirically determined mean inner-mate distance. The datasets used in our analysis of the three cell lines are available in the Additional files 2, 3 and 4, respectively.

### CE annotations and inclusion analysis

All CE events were obtained from the annotation files provided by Katz *et al.* [27]. The annotation files (release 2) included all alternative splicing events that were derived from the inventory of human genes and mRNA isoforms published by Nilsen *et al.* [44]. We made use of this catalog to get the average length of the human cassette exons. There are in total 42485 human cassette exons in release 2 and the average length is 146.17 bp.

RNA-seq data were analyzed by MISO [27], a statistical model that estimates the expression of alternatively spliced exons and isoforms, and assesses confidence in these estimates, to determine the inclusion level of each CE. In particular, MISO outputs the “percentage spliced in” (PSI or *ψ*) that denotes the fraction of mRNA isoforms including CEs of interest. On the basis of *ψ*, we partitioned the CE samples into different bins. For example, we define the 0.75–0.25 partitioning rule as that the high portion consists of CEs with inclusion level greater than 0.75, the low portion consists of those with inclusion level less than 0.25 and the middle portion consists of the rest ones. After removal of those skipping exons whose hosting genes are not expressed or counts of mapped short reads are too rare to compute the inclusion ratio with statistical significance (MISO parameter *min*_*event*_*reads* is set to 20), there are 14762, 17142 and 16052 skipping exons for Gm12878, K562 and H1-hESC, respectively.

### Differentially expressed CE detection

We used MISO [27] to detect differentially expressed CEs between samples. After the calculation of CE inclusion levels, MISO computes the *Bayes factor*, which represents the weight of the evidence in the data in favor of differential inclusion, for each exon between two samples. In particular, the Gm12878 and K562 include 2 RNA-seq replicates, while H1-hESC includes 4 RNA-seq replicates. We carried out differentially expressed CE detection for each pair of replicates, each of which came from two different cell lines. Therefore, we have 4 pairs of replicate-replicate comparisons for Gm12878 vs. K562, and eight pairs of replicate-replicate comparisons for both Gm12878 vs. H1-hESC and K562 vs. H1-hESC. After differentially inclusion detection, differentially expressed CEs were filtered out based on their coverage or magnitude of change. We filtered out CE samples by such parameter setting: *Bayes*_*factor* = 2 and *delta* - *psi* = 0.15. Finally, we performed intersect operator on all replicate-replicate comparisons of each pair of cell lines, and got the differentially expressed CEs. The numbers of differentially expressed CE samples are shown in Table 1. The differentially expressed CE samples are listed in Additional file 5.

### HM signal calculation

HM signals on CE and flanking regions were calculated in two steps. First, the smoothed read counts falling into the regions of interest were added up. If only part of the smoothing windows overlaps the regions of interest, the read counts were allocated in proportion to the fraction of overlapping part of the smoothing window. Second, the total read count was divided by the number of base pairs included in the computed region to obtain the mean signal strengths of HMs over the target region.

### Logistic regression

Logistic regression is a type of widely used probabilistic statistical classification model that measures the relationship between a categorical response variable and one or more independent explanatory variables. The advantage of logistic regression over other classifiers such as SVM is the interpretability of the regression results, that is, the regression coefficient and odds-ratio of each explanatory variable reflect its strength of correlation to the response variable. Therefore, we selected logistic regression to model the relationship between HMs and CE inclusion. Specifically, logistic regression can be formulated as below:
1

in which *x*_*i*_ represents a certain type of histone modification, *y* represents the CE inclusion level and *β*_*i*_ is the regression coefficient.

We employ the standard software SAS 9.2 to conduct logistic regression analysis. For SAS’s logistic procedure, we adopt the likelihood score criterion for variable selection, i.e., *χ*^2^ values were calculated to evaluate the model to choose a new variable that achieves the highest *χ*^2^ value compared to other remaining variables not included the current model (i.e., *best* parameter is set to 1). The likelihood score curves for the three cell lines were shown in Additional file 1: Figure S8, from which we found that the chi-square score increases rapidly at the beginning and then tends to steady after about 10 variables were included in the model.

To get statistical robust model, we randomly extracted 1500 samples from each classes of samples after CE inclusion level discretization by adopting the SAS’s *surveyselect* process, so as to eliminate the effect of imbalanced number of training samples that are generated by different discretization thresholds *δ*. We thus obtained a dataset including 3000 CE samples, and next randomly extract two thirds of these 3000 samples as training set to build the logistic regression model and the remaining as an independent test set. Furthermore, the random sampling, training and test procedures were repeated 50 times to get the mean performance measures of the logistic regression model. For the experiments of predicting differentially expressed CEs across cell types, we used all samples and run the same process to build statistically significant models, as the numbers of differentially expressed CE samples are no more than 1500. The SAS code used in our analysis is available in Additional file 6.

## Electronic supplementary material

Additional file 1: **Supplementary Figures S1-S17 referred to in this paper** (PDF 5 MB)

Additional file 2: **Datasets of Gm12878 in SAS data format.** (ZIP 3 MB)

Additional file 3: **Datasets of K562 in SAS data format.** (ZIP 6 MB)

Additional file 4: **Datasets of H1-hESC in SAS data format.** (ZIP 3 MB)

Additional file 5: **Differentially expressed CE samples across three cell lines.** (ZIP 49 KB)

Additional file 6: **SAS source codes in our analysis.** (ZIP 7 KB)
