# Supplementary material for: Histone modifications involved in cassette exon inclusions: a quantitative and interpretable analysis
Source: BMC Genomics. 2014 Dec 19;15(1):1148. doi: 10.1186/1471-2164-15-1148 (PMC4378014; doi:10.1186/1471-2164-15-1148)
Supplement: Supplementary file 1 — Additional file 1: Supplementary Figures S1-S17 referred to in this paper (PDF 5 MB) [file 12864_2014_6886_MOESM1_ESM.pdf]

Supplementary files

October 24, 2014

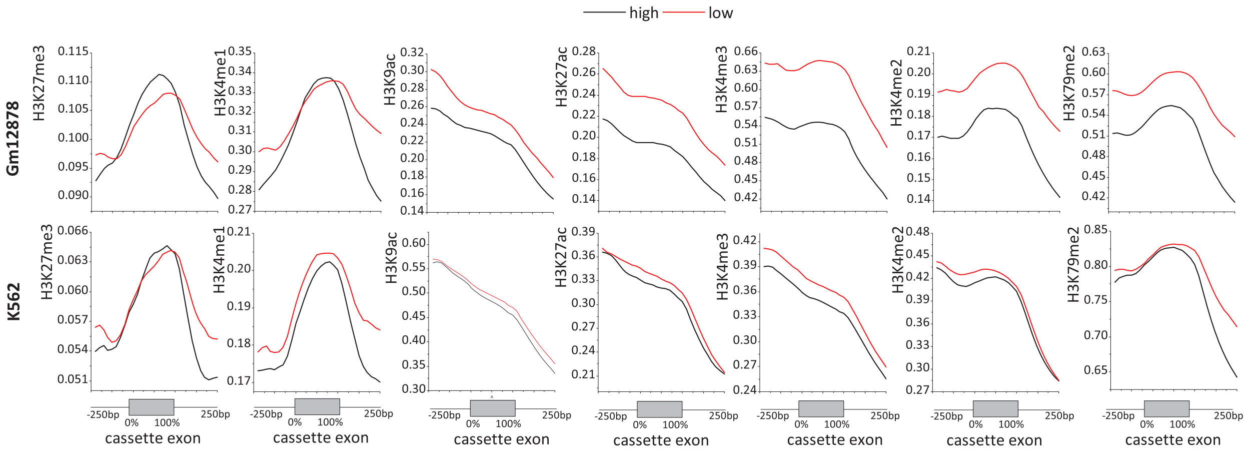

Figure S1: Mean ChIP-seq signal distributions of seven type of HM on CEs and ±250bp flanking regions. The ChIP-seq signals were averaged over each portion of CE samples, which were partitioned into high and low portions according to inclusion levels. The upper and lower half parts correspond to the signal distributions for Gm12878 and K562, respectively.

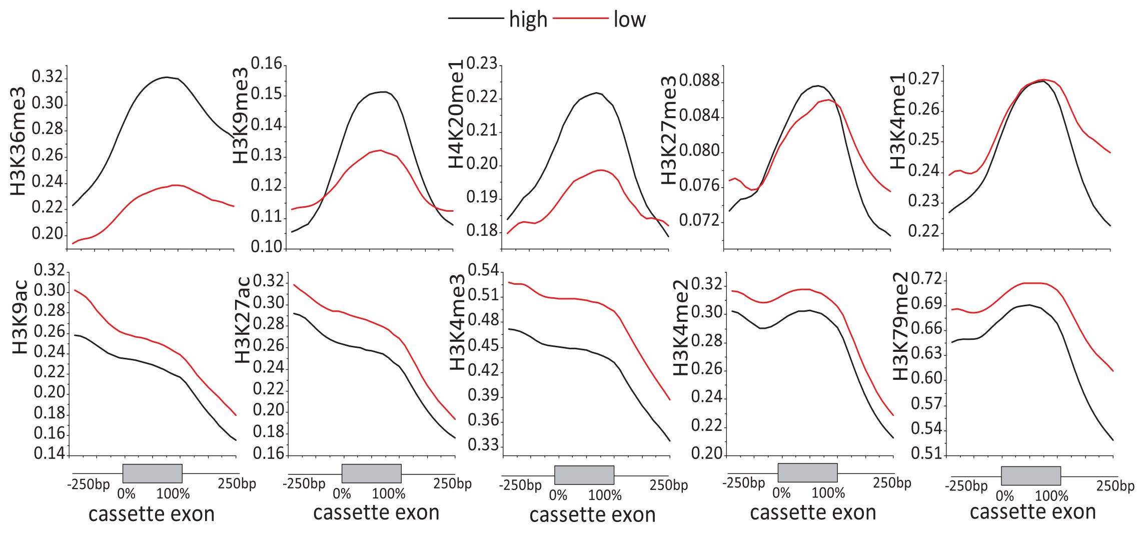

Figure S2: Mean ChIP-seq signal distributions of ten type of HM on CEs and ±250bp flanking regions for H1-hESC cell lines. The ChIP-seq signals were averaged over each portion of CE samples, which were partitioned into high and low portions according to inclusion levels.

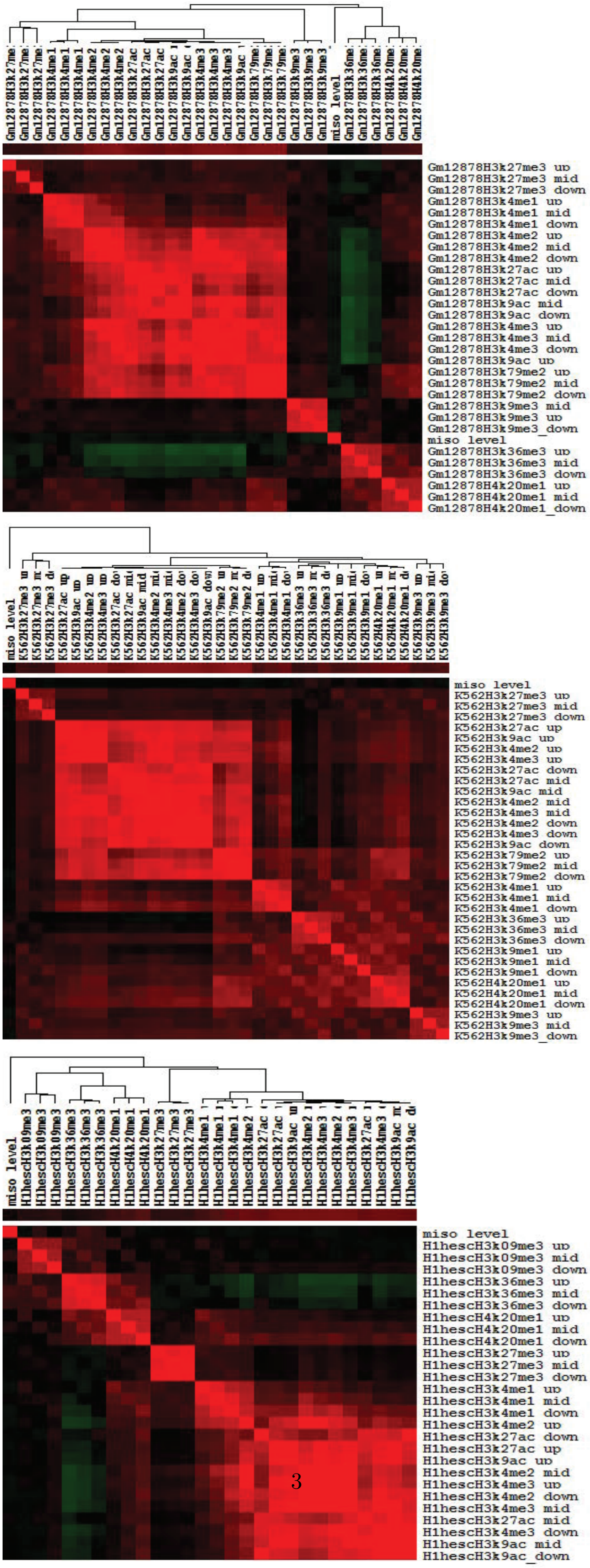

Figure S3: Heatmaps and hierarchical clusterings of the Pearson correlation coefficients between HMs, as well as the CE inclusion levels (showed as miso_level), for the three cell lines Gm12878, K562 and H1-hESC.

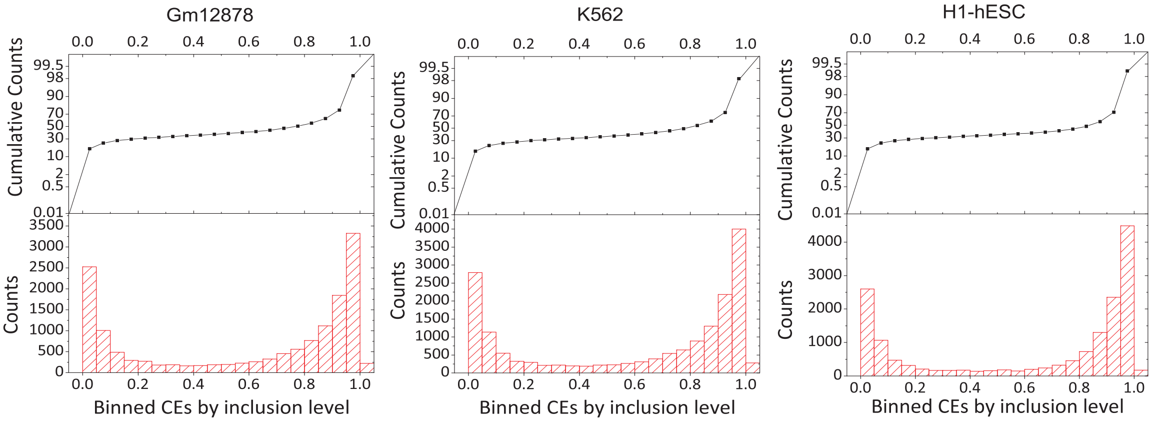

Figure S4: Histograms and cumulative distributions of CE samples binned by their inclusion levels for the three cell lines Gm12878, K562 and H1-hESC.

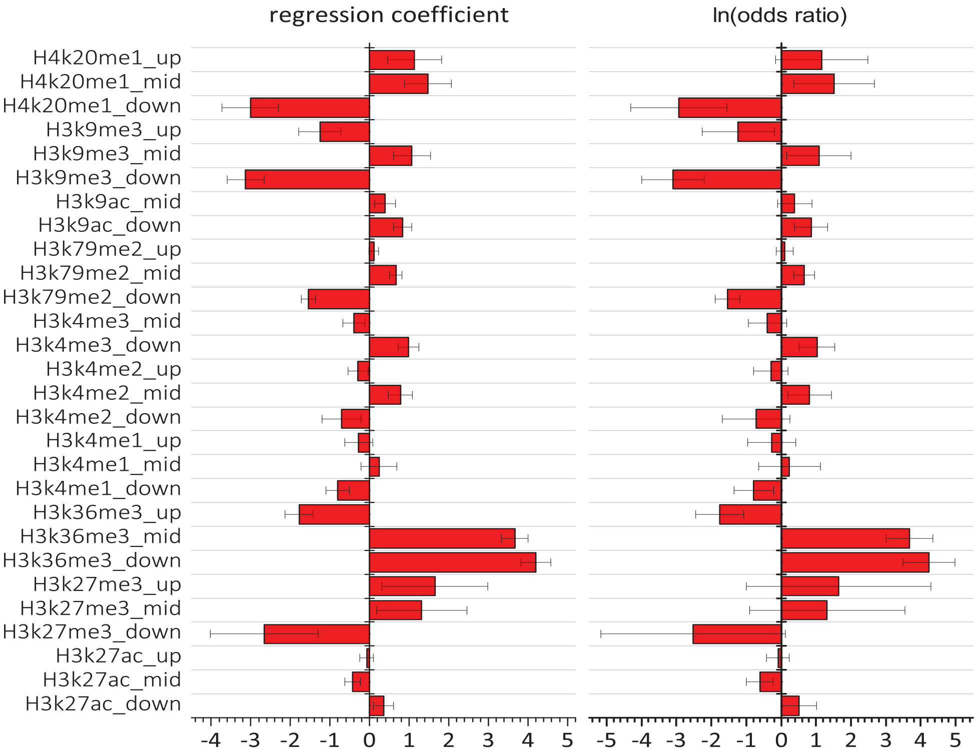

Figure S5: Logistic regression coefficients and odds ratios on natural-log scale for each component of ten type of HMs for Gm12878 cell line. The suffixes _mid, _up and _down of certain type HM represent the three components corresponding to the ChIP-seq signals on the CE, upstream and downstream flanking regions, respectively. The CE inclusion levels are discretized by setting δ to 0.85.

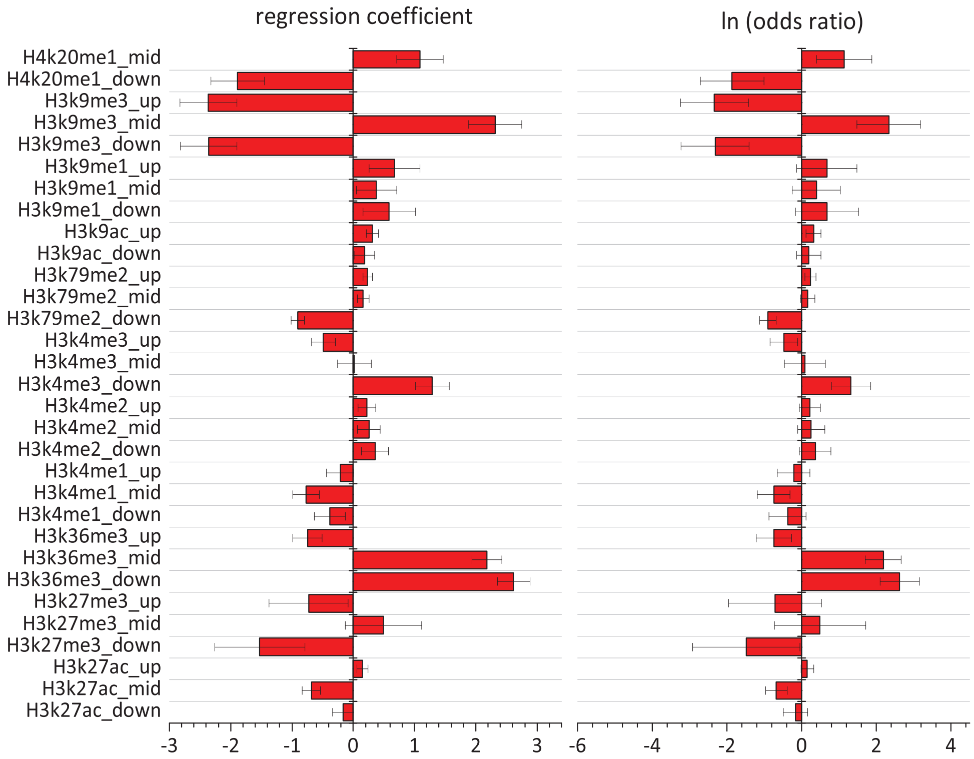

Figure S6: Logistic regression coefficients and odds ratios on natural-log scale for each component of ten type of HMs for K562 cell line. The suffixes _mid, _up and _down of certain type HM represent the three components corresponding to the ChIP-seq signals on the CE, upstream and downstream flanking regions, respectively. The CE inclusion levels are discretized by setting δ to 0.85.

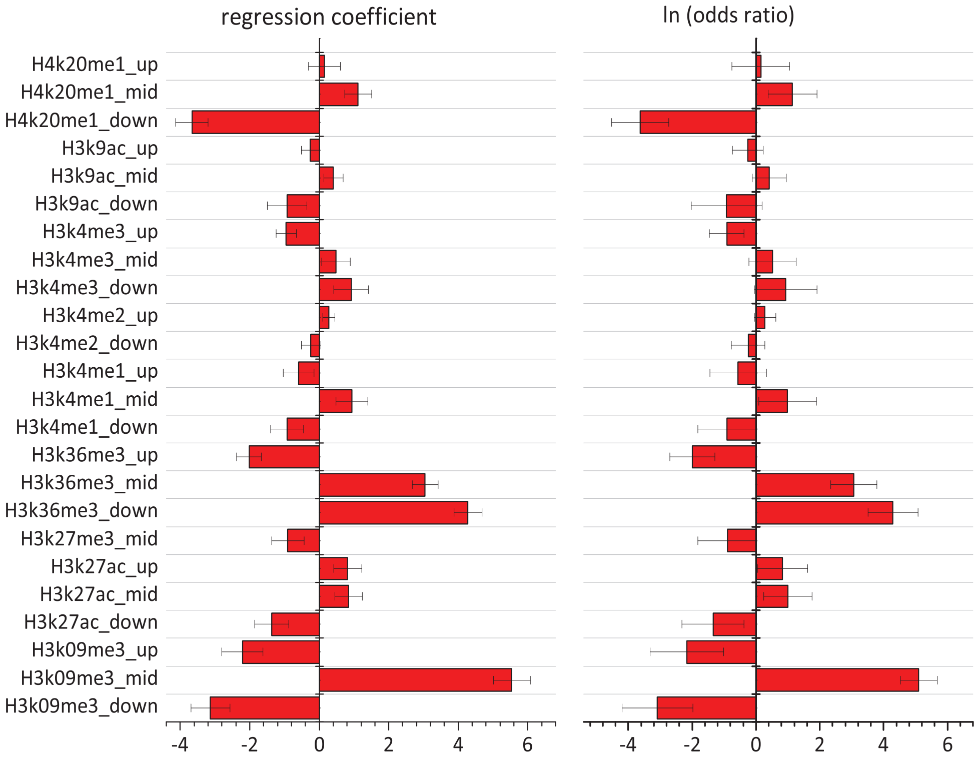

Figure S7: Logistic regression coefficients and odds ratios on natural-log scale for each component of ten types of HMs for H1-hESC cell line. The suffixes _mid, _up and _down of certain type HM represent the three components corresponding to the ChIP-seq signals on the CE, upstream and downstream flanking regions, respectively. The CE inclusion levels are discretized by setting δ to 0.85.

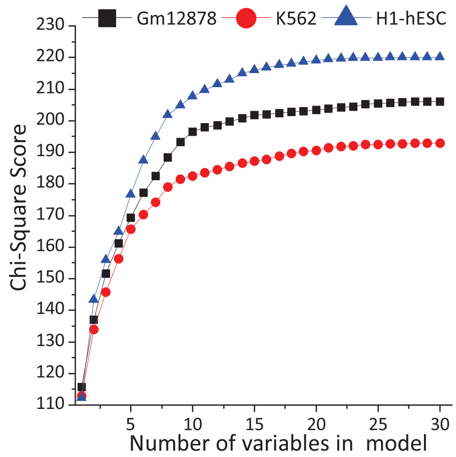

Figure S8: Chi-square score curves for evaluating the goodness of fitting to data by the logistic regression models. The models were progressively built and each time a new variable was added into the current model to evaluate the degree of fitting to data.

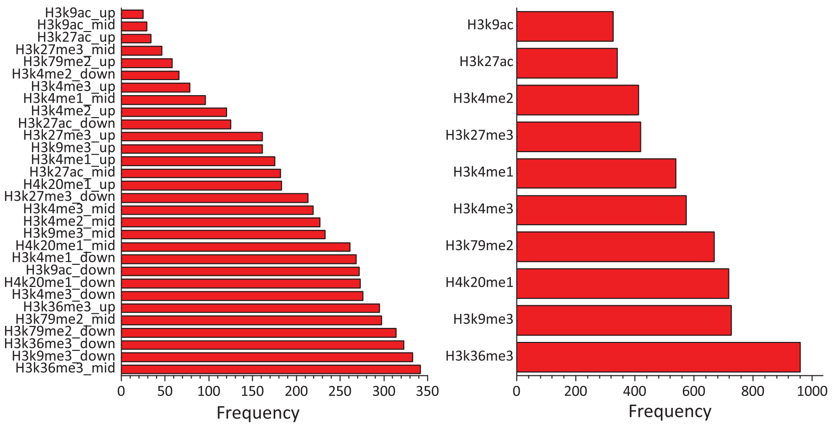

Figure S9: The frequencies of each HMs included in the top 10 logistic regression models that were repeatedly built for 50 times. The training set includes 2/3 CE samples randomly sampled from original 14,762 CEs of GM12878. Each component of HMs included in the top 10 models were taken into account, and the sampling and learning process were repeated 50 times to calculate the total frequency of each component of the HMs.

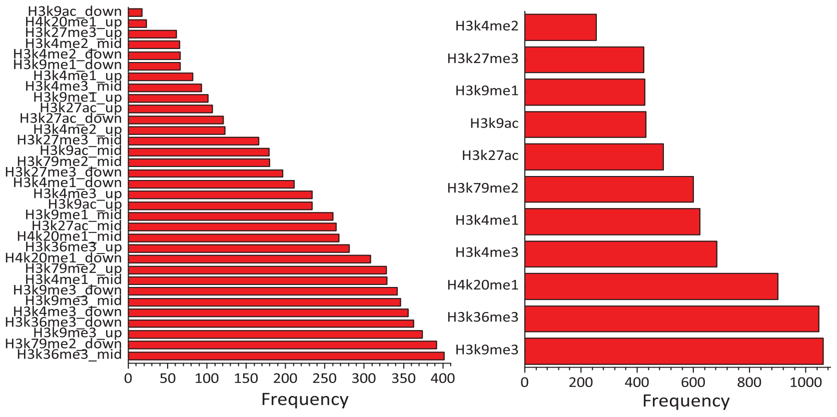

Figure S10: The frequencies of each HMs included in the top 10 logistic regression models that were repeatedly built for 50 times. The training set includes 2/3 CE samples randomly sampled from original 17,142 CEs of K562. Each component of HMs included in the top 10 models were taken into account, and the sampling and learning process were repeated 50 times to calculate the total frequency of each component of the HMs.

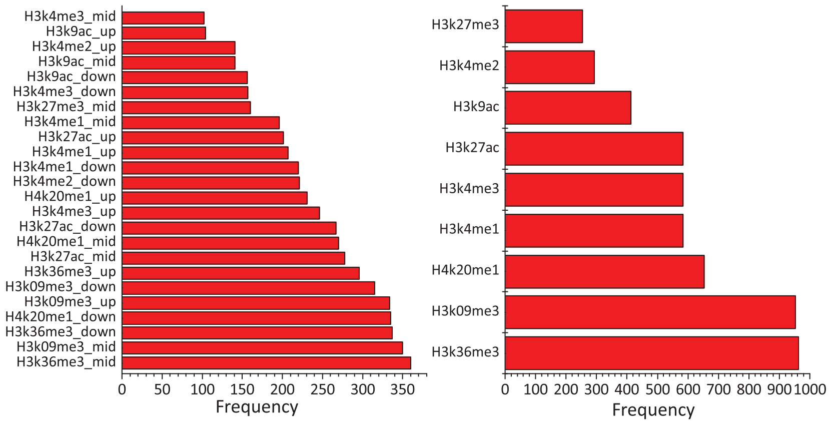

Figure S11: The frequencies of each HMs included in the top 10 logistic regression models that were repeatedly built for 50 times. The training set includes 2/3 CE samples randomly sampled from original 16,052 CEs of H1-hESC. Each component of HMs included in the top 10 models were taken into account, and the sampling and learning process were repeated 50 times to calculate the total frequency of each component of the HMs.

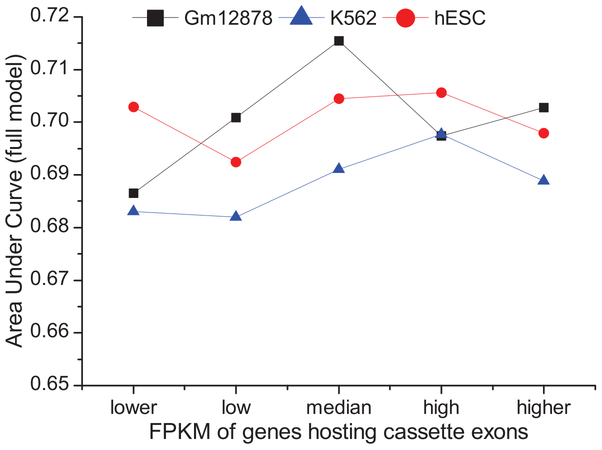

Figure S12: AUC values of the logistic regression models built on five subset of training sets generated by equally partitioning the CE samples into five subsets according to their FPKM values for Gm12878, K562 and H1-hESC, respectively.

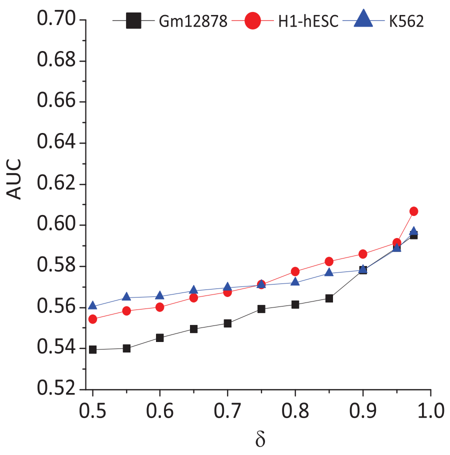

Figure S13: AUC curves of the logistic regression models built based on the HM signals excluding the three types of HMs, H3K36me3, H3K9me3 and H4K20me1, for Gm12878, K562 and H1-hESC, respectively.

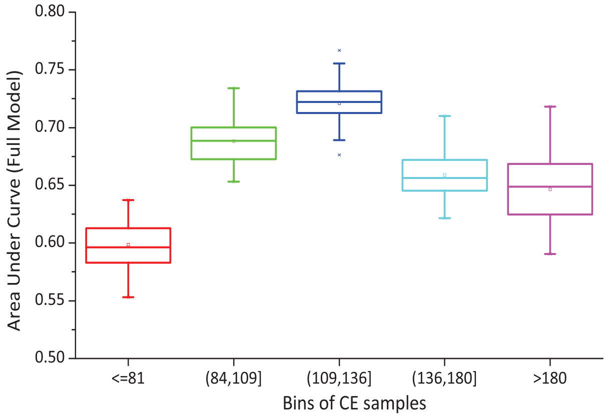

Figure S14: AUC boxplots of the logistic regression models learned from five subsets of CE samples of K562. All CEs were ranked in ascending order according to the exon lengths and then split into five bins by equal-depth partitioning rule.

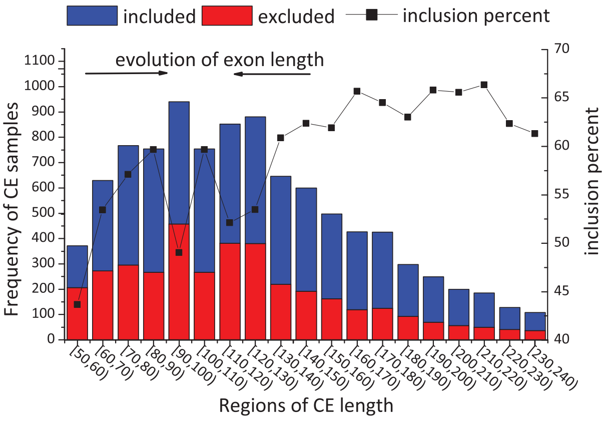

Figure S15: Histogram of the CE frequency over CE length for K562, together with the percent of CEs included in mature RNAs.

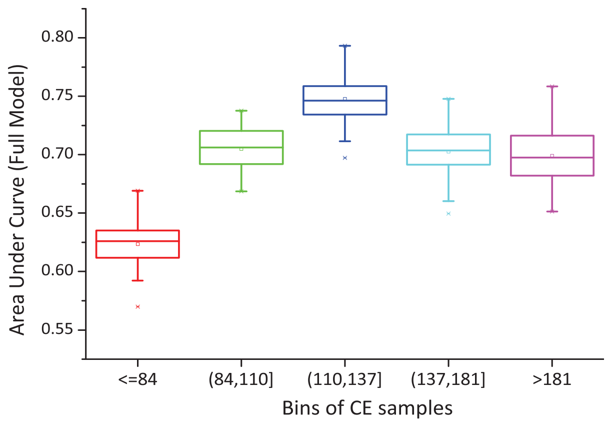

Figure S16: AUC values of the logistic regression models learned from five subsets of CE samples of H1-hESC. All CEs were ranked in ascending order according to the exon lengths and then were split into five bins by equal-depth partitioning rule.

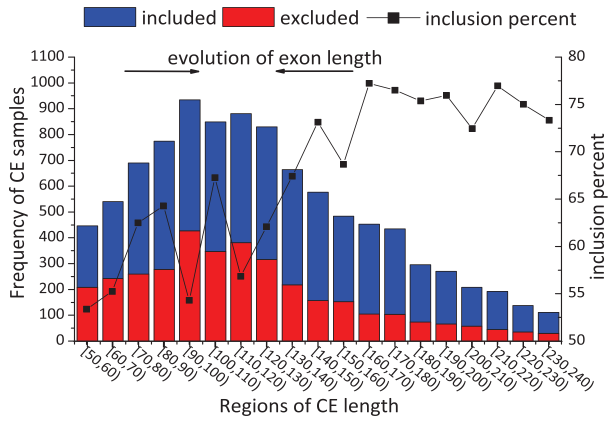

Figure S17: Histogram of the CE frequency over CE length for H1-hESC, together with the percent of CEs included in mature RNAs.

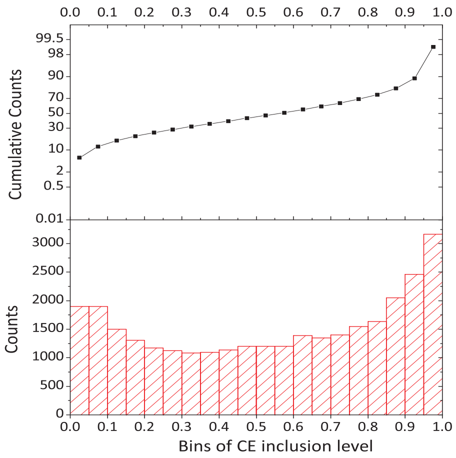

Figure S18: Histograms and cumulative distributions of CE samples binned by their inclusion levels for the three cell lines for CD4+T cell. The RNA-seq data was obtained from GEO (GSM406414) published published by Chepelev *et al.*, (Nucleic Acids Res., 2009).

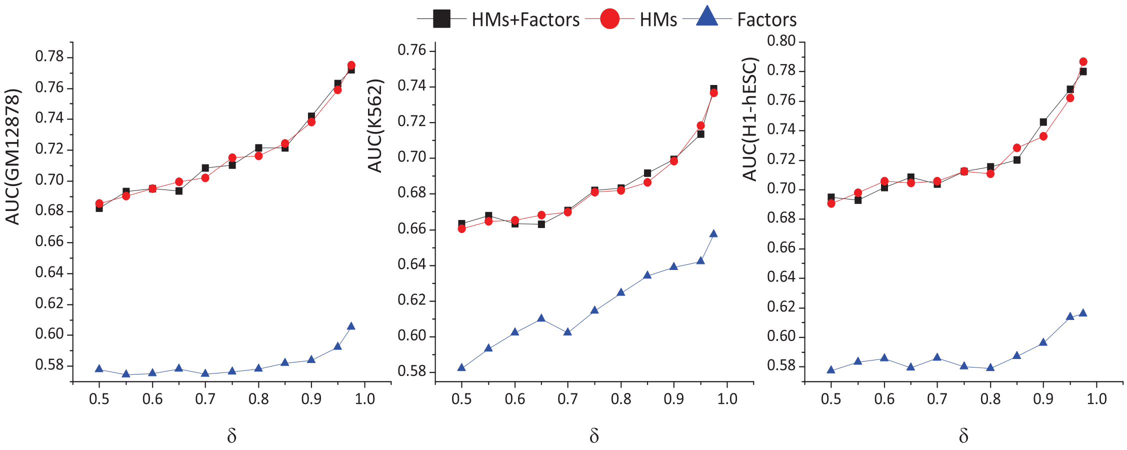

Figure S19: AUC curves of the logistic regression models built independently on the HMs, protein factors, both HMs and other protein factors, respectively.
